# Outcome data for the remote patient monitoring over three years of over 1000 patients in Northern Ireland with a long-term chronic illness

**Published:** 2012-06-15

**Authors:** Peter Edward Range

**Affiliations:** Alere Connected Health Limited, UK

**Keywords:** outcome-data, managed-service, transformation, innovation, database

## Abstract

Over a period of four years Alere Connected Health’s (ACH) management and specialist telehealth nurses have designed, implemented and delivered a completely new and re-engineered model of community care for people with long-term conditions (LTC) that has resulted in the delivery of a full ‘end to end’ clinical telehealth managed service monitoring over the period for over 1000 patients from two NHS Trusts in Northern Ireland. ACH were commissioned to develop programmes to deliver chronic disease monitoring and from the outset it was clear that the Department of Health was looking for a ‘service delivery’ partner rather than a ‘kit provider’. This regional telehealth programme in partnership with Belfast Health and Social Care Trust and South Eastern Health and Social Care Trust managed patients with one or more long-term conditions, their main chronic conditions being COPD, CHF and Diabetes. This new model of care has now been commissioned by the NHS in Northern Ireland as a National telehealth monitoring service awarding a 6-year contract for the monitoring of several thousand people with a LTC. The ACH telehealth service is ‘technology agnostic’ and can be used with point of care devices from any manufacturer, making it easier for commissioners and users that are already familiar with a particular telehealth product or manufacturer.

During the four-year programme the ACH team developed an innovative web based activity database and telehealth management system software (TMS) in order to gather additional data from and about the patients care in order to determine more accurate programme evaluation data. The data gathered involved clinical, technical and statistical information in order to determine patient utilisation, return on investment (ROI) information and programme outcomes not usually derived from most commercial telehealth ‘back-end’ web server solutions. (In the process the ACH nursing team won two National Awards for ‘Innovation and Partnering’ with the NHS.) A number of significant outcomes and goals have been achieved during the past four years about service delivery and clear evidence suggests that telehealth home patient monitoring can be used as a method of reducing hospital admissions, it appears to promote patients to become better self managers of their long term condition and ultimately enjoy a better quality of life, whilst delivering cost efficiencies to the health care provider. This resulted in fewer unnecessary appointments with their GP.

Patients have reported high levels of satisfaction with the service and health providers are happy that they have been able to concentrate their attention on the patients that really need them. All in all it’s a “win-win situation”. Some of the programme findings and outcome data are as follows:

- 60% saving of COPD patient unplanned hospital admissions
- 33% saving of CHF patient unplanned hospital admissions
- 32% of patients escalated during ‘out of hours’
- After a period of three years telehealth monitoring of (n=766 COPD patients) equalling some 3% of the Northern Ireland COPD population, the unplanned hospital admission rate for COPD patients reduced from 42% to 9%.

